# HER2 and hormone receptor conversion after neoadjuvant therapy for breast cancer

**DOI:** 10.3389/fonc.2025.1522460

**Published:** 2025-06-09

**Authors:** Jing Wang, Xin Long, Mingxi Tang, Xiuli Xiao

**Affiliations:** ^1^ Department of Pathology, Yaan People’s Hospital, Yaan, Sichuan, China; ^2^ Department of Pathology, The Affiliated Hospital of Southwest Medical University, Luzhou, Sichuan, China; ^3^ School of Basic Medical Sciences, Southwest Medical University, Luzhou, Sichuan, China; ^4^ School of Clinical Medical Sciences, Southwest Medical University, Luzhou, Sichuan, China

**Keywords:** breast cancer, neoadjuvant therapy, estrogen receptor, progesterone receptor, human epidermal growth factor receptor 2

## Abstract

**Background:**

The expression of estrogen receptor (ER), progesterone receptor (PR), and human epidermal growth factor receptor 2 (HER2) in residual lesions may be different compared with primary tumors of the breast after neoadjuvant therapy (NAT). Given the clinical implications of hormone receptor expression for breast cancer management, we assessed conversions in ER, PR, and HER2 in breast cancer patients after NAT.

**Methods:**

Our study comprised 589 individuals with aggressive breast cancer who underwent NAT. We examined the ER, PR, and HER2 statuses in primary and residual breast cancers and investigated the relationship between receptor conversion and clinicopathological variables.

**Results:**

The pathologic complete response (pCR) rate for the overall cohort was 38.7%, with pCR rates of 57.0%, 13.1%, and 33.3% for HER2-positive, Luminal, and triple-negative breast cancer (TNBC), respectively. Cases with negative hormone receptor expression were more likely to achieve pCR than positive cases. The highest pCR rates were seen in HER2-positive breast cancers, followed by HER2-zero and HER2-low tumors. After NAT, there were 26 (7.8%) cases of ER status conversion and 53 (16.0%) cases of PR status conversion. The conversion of hormone receptors was mainly from positive to negative. When cases were categorized as HER2-negative or positive, 15 (5.1%) cases had a conversion of HER2 status, predominantly positive to negative. When cases were classified as HER2-zero, -low, or -positive, HER2 status conversion happened in 54 (18.6%) cases and was mostly happened between HER2-zero and HER2-low. HER2 status before NAT correlated with ER and HER2 conversion.

**Conclusion:**

Some breast cancer patients may show ER, PR, or HER2 status conversion after NAT. Residual lesions need to be immunohistochemically re-tested to reassess the patient’s receptor expression status and to adjust the subsequent treatment regimen.

## 1 Introduction

According to data released by the International Agency for Research on Cancer, approximately 2.3 million new cases of breast cancer were reported globally in 2022, ranking first in the incidence of female malignant tumors (1). Breast cancer is a systemic disease that is usually treated with surgery, radiotherapy, chemotherapy, immunotherapy, endocrine therapy, and targeted therapy (2). Neoadjuvant therapy (NAT) is a systemic adjuvant therapy administered prior to surgical resection of the neoplasm that aims to reduce the size of the primary lesion, decrease axillary staging, improve breast retention, determine drug sensitivity, and guide subsequent treatment and prognostic analysis (3).

Prior to NAT, ultrasound-guided core needle biopsy (CNB) is commonly used to obtain the status of estrogen receptor (ER), progesterone receptor (PR), human epidermal growth factor receptor 2 (HER2), and Ki-67 in the lesions of patients with breast cancer. Based on receptor expression, breast cancers are classified into the following four molecular subtypes: Luminal A, Luminal B, HER2-overexpressing, and triple-negative breast cancers (TNBC) (4). The pathologic complete response (pCR) rate is a major factor in response to NAT efficacy, and pCR is used as an early surrogate endpoint for predicting overall survival (OS) (5). Breast cancer patients who achieve pCR after NAT have a better prognosis (6). With advances in technology and medicine, systemic therapy has been developed, and the percentage of patients with pCR after NAT has increased significantly (7). However, some patients still have residual lesions after NAT. Determining the molecular subtype of the remaining tumor foci following NAT in non-pCR patients is crucial for prognosis and treatment regimen optimization (8).

Owing to the highly heterogeneous nature of breast cancer, preoperative CNB may not fully reveal the true nature of the neoplasm. ER, PR, and HER2 expression in residual lesions may be different compared with the primary tumors after NAT (9–11). In the subset of patients with receptor status conversion, diagnostic strategies and the effect on prognosis are controversial. Receptor conversion has a prognostic value and may influence clinicians’ therapeutic decisions. As a result, this study retrospectively examined variations in ER, PR, and HER2 expression in 589 breast cancer patients treated with NAT to assess the effect of NAT on the status of these biomarkers and to investigate the clinicopathological causes of these variations.

## 2 Materials and methods

### 2.1 Patient selection

Clinical and pathological data on invasive breast cancer patients treated at the Southwest Medical University Affiliated Hospital in Sichuan Province between January 2019 and June 2023 were collected. Inclusion criteria included female breast cancer patients with primary invasive cancer diagnosed by CNB prior to treatment, with at least four cycles of NAT and complete clinical and pathological data. Male patients or those with bilateral breast cancer, radiological evidence of distant metastases, a history of additional malignancies, prior endocrine treatment, tumor-targeted radiation, or local excision were excluded from the study. pCR is defined as the absence of residual invasive cancer in the completely resected breast specimen and all sampled regional lymph nodes (ypT0/Tis ypN0 according to AJCC staging criteria) (12).

### 2.2 Immunohistochemistry staining interpretation and grouping standards

Immunohistochemistry (IHC) was performed to assess ER, PR, HER2, and Ki-67 status in pre-treatment CNB and surgical resection specimens. IHC were detected using the Roche Ventana Benchmark automated IHC system. The detection of ER, PR, and HER2 was performed using antibodies from Roche (ER clone: SP1; PR clone: 1E2; HER2 clone: 4B5). The Ki-67 antibody (clone: MIB.I) was provided by Beijing Zhongshan Golden Bridge Biotechnology Co., Ltd. ER/PR tumor cell nuclear staining <1% indicated ER/PR negativity and ≥1% indicated positivity. Hormone receptor (HR) positivity was defined as ER or PR positivity (13).

. According to the American Society of Clinical Oncology/College of American Pathologists (ASCO/CAP) guidelines (14), HER2 IHC staining results were graded as follows: HER2 0: no staining or ≤10% of invasive carcinoma cells show incomplete, weak cell membrane staining; HER2 1+: >10% of invasive carcinoma cells show incomplete, weak cell membrane staining; HER2 2+: >10% of invasive carcinoma cells show complete, weak to moderately intense cell membrane staining or ≤10% of invasive carcinoma cells show strong and complete cell membrane staining; and HER2 3+: >10% of invasive cancer cells show strong, complete, and uniform cell membrane staining. Fluorescence *in situ* hybridization (FISH) was performed to detect HER2 gene amplification in IHC 2+ tumors, utilizing the HER2 gene amplification kit from Beijing Jinpujia Company.HER2-positive was defined as IHC 3+ or IHC 2+ with FISH amplification. HER2-low was defined as IHC 1+ or IHC 2+ without FISH amplification, and HER2-zero tumors were IHC 0. HER2-negative comprised HER2-zero and HER2-low tumors. The percentage of tumor nuclei stained in the examined IHC sections was used to compute the Ki67 expression levels. The International Ki67 in Breast Cancer Working Group consensus is that Ki-67 <5% or >30% can be used to estimate prognosis (15). Therefore, in this study, 15% was used as a threshold to categorize Ki-67 into low expression (Ki-67 <15%) and high expression (Ki-67 ≥15%).

The following categories were applied to the cases based on their IHC status: Luminal (HR-positive and HER2-negative); HER2-overexpressing (HER2-positive, regardless of HR status); and TNBC (HR-negative and HER2-negative).

### 2.3 Statistical analysis

SPSS 25.0 was used for data processing and analysis. The count data are displayed as the total number of instances and the percentage (%). Pearson’s chi-square test and Fisher’s exact test were used for group comparisons. Cohen’s kappa test was used in the consistency analysis. *P* < 0.05 was considered statistically significant.

## 3 Results

### 3.1 Patient cohorts and clinicopathologic features

We collected clinical and pathological data from 589 breast cancer patients who underwent NAT. **Table 1** illustrates the major clinicopathological characteristics of the entire cohort. The median age was 51 (29–78) years old. In CNB specimens, the majority of patients had invasive ductal carcinoma (n = 556, 94.4%) with a histologic grade of 2 (n = 466, 79.1%). In most patients, the maximum diameter of the tumor before treatment was 2–5 cm (n = 434, 73.7%). The immunophenotypes of all cases before treatment were as follows: Luminal 29.9% (n = 176), HER2-overexpressing 48.2% (n = 284), and TNBC 21.9% (n = 129). Nearly half of the patients received anti-HER2 targeted therapy (n = 277, 47.0%).

**Table 1:** Main clinicopathologic characteristics.

Characteristics	N	%
Age (years)		
<50	271	46.0
≥50	318	54.0
Menopausal status		
Pre-menopausal	278	47.2
Post-menopausal	311	52.8
Histology		
Invasive ductal carcinoma	556	94.4
Other (Special type)	33	5.6
Histological grade		
1	11	1.9
2	466	79.1
3	112	19.0
Pretreatment tumor size(cm)		
≤2	65	11.0
2~5	434	73.7
≥5	90	15.3
Lymph node metastasis		
Yes	390	66.2
No	199	33.8
Ki-67		
<15%	41	7.0
≥15%	548	93.0
Molecular subtypes of primary tumors		
Luminal	176	29.9
HER2-overexpressing	284	48.2
TNBC	129	21.9
Anti-HER2 targeted therapy		
Yes	277	47.0
No	312	53.0
Miller-Payne classification		
1	13	2.2
2	67	11.4
3	169	28.7
4	104	17.6
5	236	40.1

### 3.2 Rate of pCR

**Figure 1:**
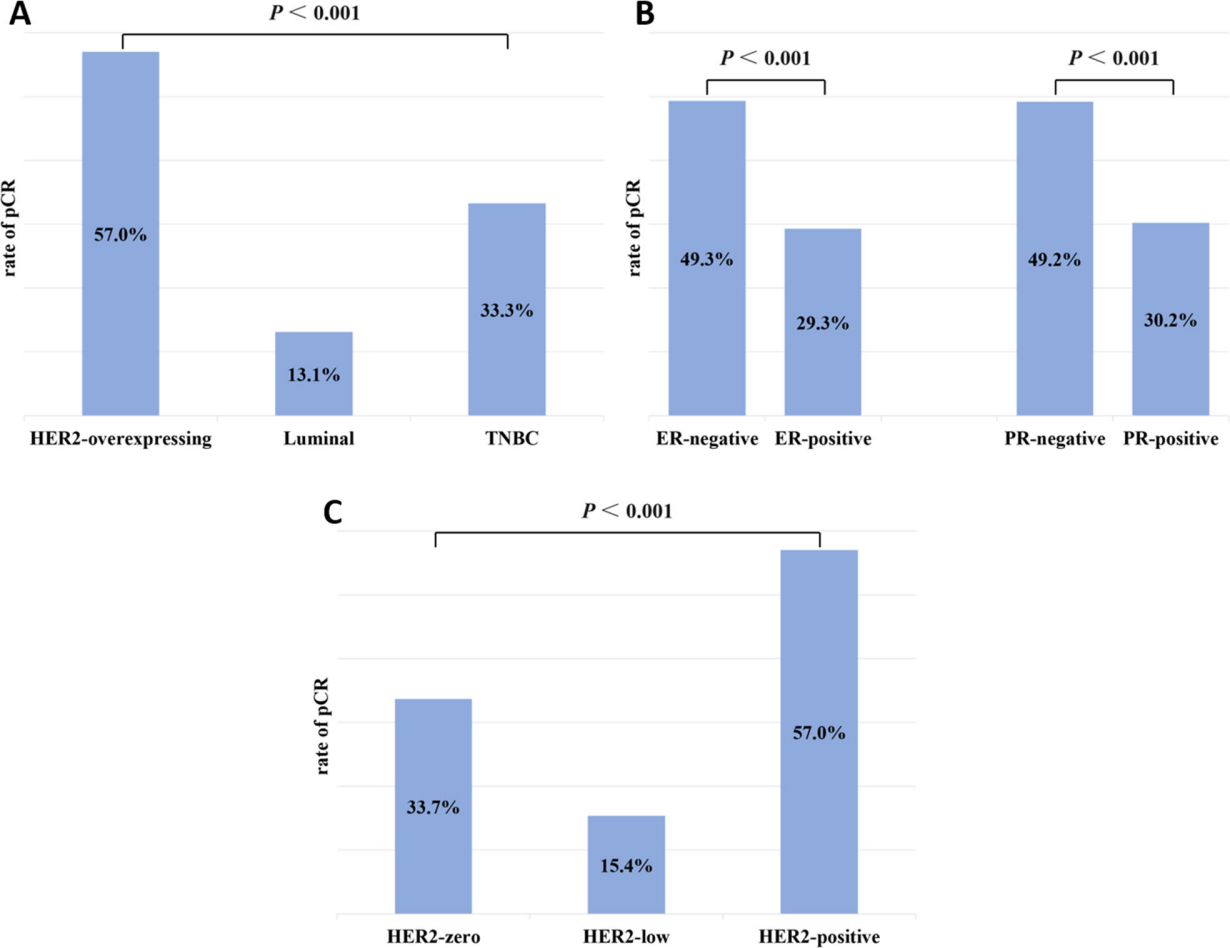
Rates of pCR by **(A)** subtype (HER2-overexpressing, Luminal, and TNBC); **(B)** hormone receptor status (ER-negative and ER-positive; PR-negative and PR-positive); and **(C)** HER2 status (zero, low, and positive).

Of the 236 patients with Miller-Payne (MP) grade 5, intravascular thrombus was detected in the specimen after surgery in one patient, and invasive cancer cells were detected in axillary lymph nodes after surgery in seven patients. Therefore, pCR was achieved in 228 cases, and the pCR rate for the overall population was 38.7% (228/589). The rate of pCR was 13.1% (23/176) for Luminal cancer, 57.0% (162/284) for HER2-overexpressing cancer, and 33.3% (43/129) for TNBC (**Figure 1A**). Thus, HER2-overexpressing tumors had a higher rate of pCR than Luminal or TNBC (*P* < 0.001). Further stratification of the HER2-overexpressing cohort by HR status revealed that the pCR rate in the HER2+/HR+ group was 50.5% (101/200), significantly lower than the 72.6% (61/84) observed in the HER2+/HR− group, with a statistically significant difference (*P* < 0.001). HR-negative patients were more likely to achieve pCR than positive cases (pCR rates in ER-negative *vs*. ER-positive: 49.3% *vs*. 29.3%, *P* < 0.001; PR-negative *vs*. PR-positive: 49.2% *vs*. 30.2%, *P* < 0.001; **Figure 1B**). There was a significant association between HER2 expression and pCR rates. The highest pCR rates were found in HER2-positive breast cancers, followed by tumors with HER2-zero and HER2-low (pCR rates in HER2-positive *vs*. HER2-zero *vs*. HER2-low: 57.0% *vs*. 33.7% *vs*. 15.4%, *P* < 0.001; **Figure 1C**).

### 3.3 Conversion of ER and PR

**Figure 2:**
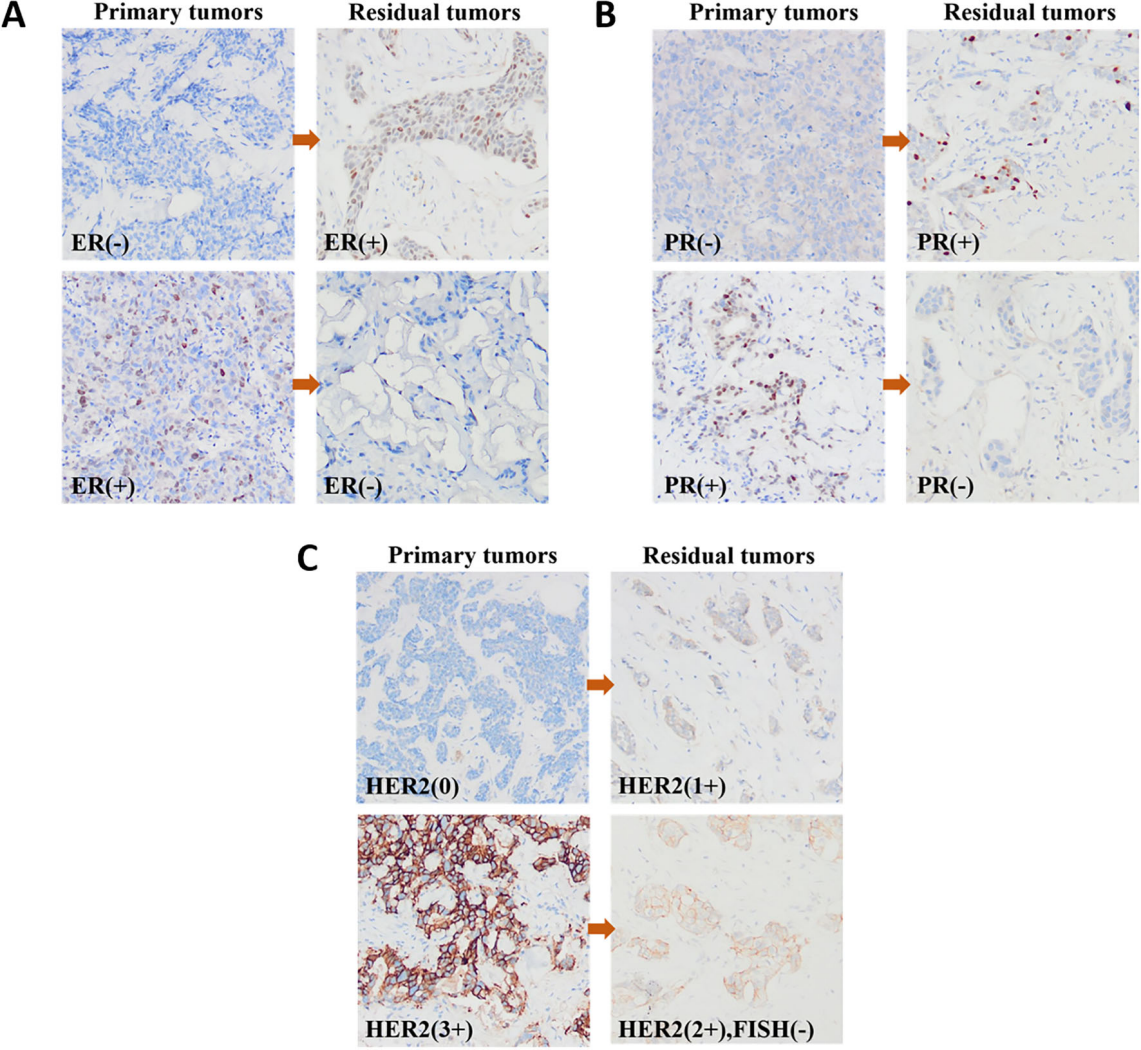
IHC images of patients with receptor conversion: **(A)** ER; **(B)** PR and **(C)** HER2.

Of the 361 non-pCR patients, 30 patients were not tested for IHC after NAT because of the low number of residual tumor cells. Therefore, 331 patients were included in the evaluation of ER and PR statuses. **Table 2** summarizes the conversion of ER and PR statuses of breast cancers from CNB specimens to residual lesions after specimen removal. **Figures 2A, B** show IHC images of patients with ER and PR conversion.

**Table 2:** Conversion of ER and PR expression between primary and residual tumors.

Primary tumors	Residual tumors	N	%
ER status			
Negative	Negative	120	36.3
	Positive	7	2.1
Positive	Negative	19	5.7
	Positive	185	55.9
PR status			
Negative	Negative	102	30.8
	Positive	17	5.1
Positive	Negative	36	10.9
	Positive	176	53.2

**Figure 3:**
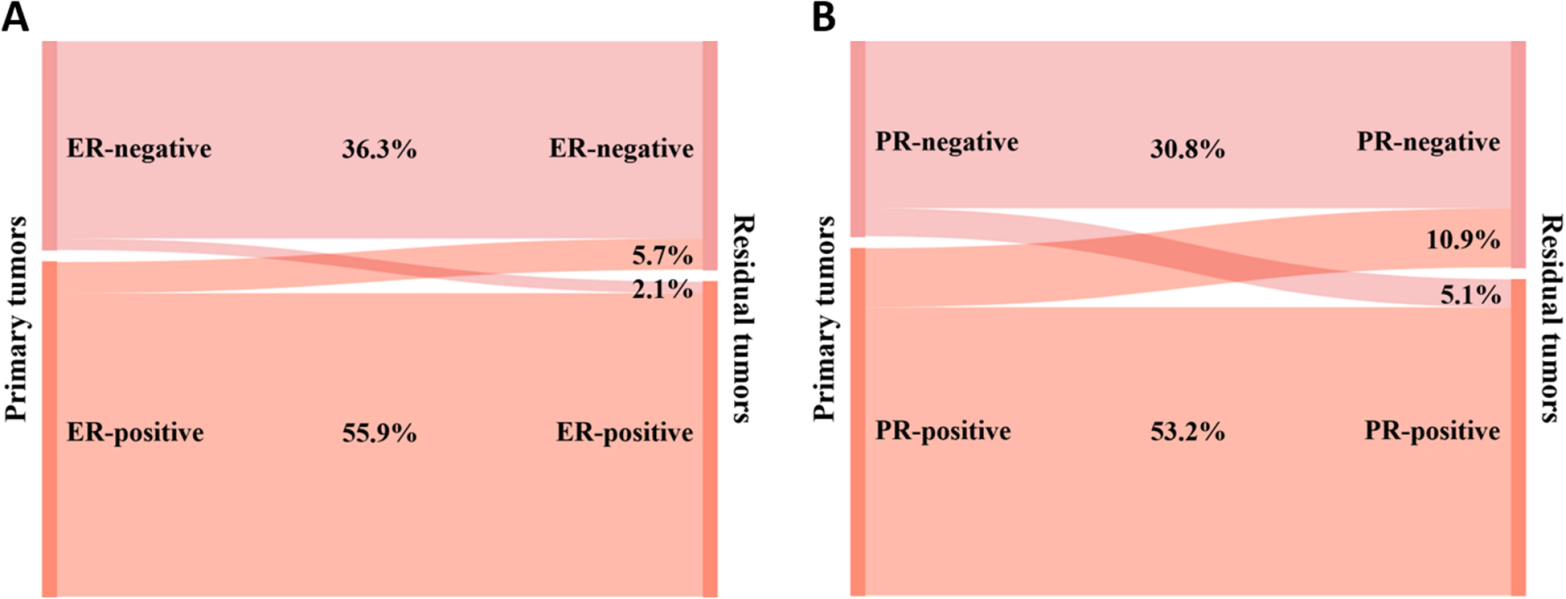
Conversion of hormone receptor expression between primary and residual tumors: **(A)** ER and **(B)** PR.

The primary breast cancer was ER-negative in 127 (38.4%) cases and ER-positive in 204 (61.6%) cases. Among residual lesions, 139 (42.0%) were ER-negative, and 192 (58.0%) were ER-positive. After NAT, 7 (2.1%) ER-negative cases converted to ER-positive, and 19 (5.7%) ER-positive cases converted to ER-negative (**Figure 3A**). The rate of ER conversion was 7.8% (n = 26), and Cohen’s kappa coefficient was 0.837, indicating that the ER status after NAT was highly consistent with that before treatment.

Among primary breast cancers, 119 (36.0%) were PR-negative, and 212 (64.0%) were PR-positive. Among the residual lesions, 138 (41.7%) cases were PR-negative, and 193 (58.3%) cases were PR-positive. As shown in **Figure 3B**, after NAT, PR-negative converted to PR-positive in 17 (5.1%) cases, and PR-positive converted to PR-negative in 36 (10.9%) cases. The PR conversion rate was 16.0% (n = 53), and Cohen’s kappa coefficient was 0.664, indicating that the consistency of PR status was relatively high.

### 3.4 Conversion of HER2

After NAT, 41 patients were HER2 IHC 2+ but did not undergo FISH testing to verify HER2 amplification status. therefore, the assessment of HER2 status included 290 patients. Primary breast cancer was HER2-negative in 205 (70.7%) cases and HER2-positive in 85 (29.3%) cases. HER2-negative cases included 143 (49.3%) HER2-low cases and 62 (21.4%) HER2-zero cases. Residual lesions were HER2-positive in 80 (27.6%) and negative in 210 (72.4%) cases. HER2-negative cases included 161 (55.5%) HER2-low cases and 49 (16.9%) HER2-zero cases. **Table 3** summarizes the HER2 status conversion of breast cancers from CNB specimens to residual lesions after specimen removal. **Figure 2C** shows IHC images of patients with HER2 conversion.

**Table 3:** Conversion of HER2 expression between primary and residual tumors.

Primary tumor	Residual tumor	N	%
Categorized as HER2 negative and positive			
Negative	Negative	200	69.0
	Positive	5	1.7
Positive	Negative	10	3.4
	Positive	75	25.9
Categorized as HER2 zero, low and positive			
Zero	Zero	36	12.4
	Low	26	9.0
	Positive	0	0.0
Low	Zero	13	4.5
	Low	125	43.1
	Positive	5	1.7
Positive	Zero	0	0.0
	Low	10	3.4
	Positive	75	25.9

**Figure 4:**
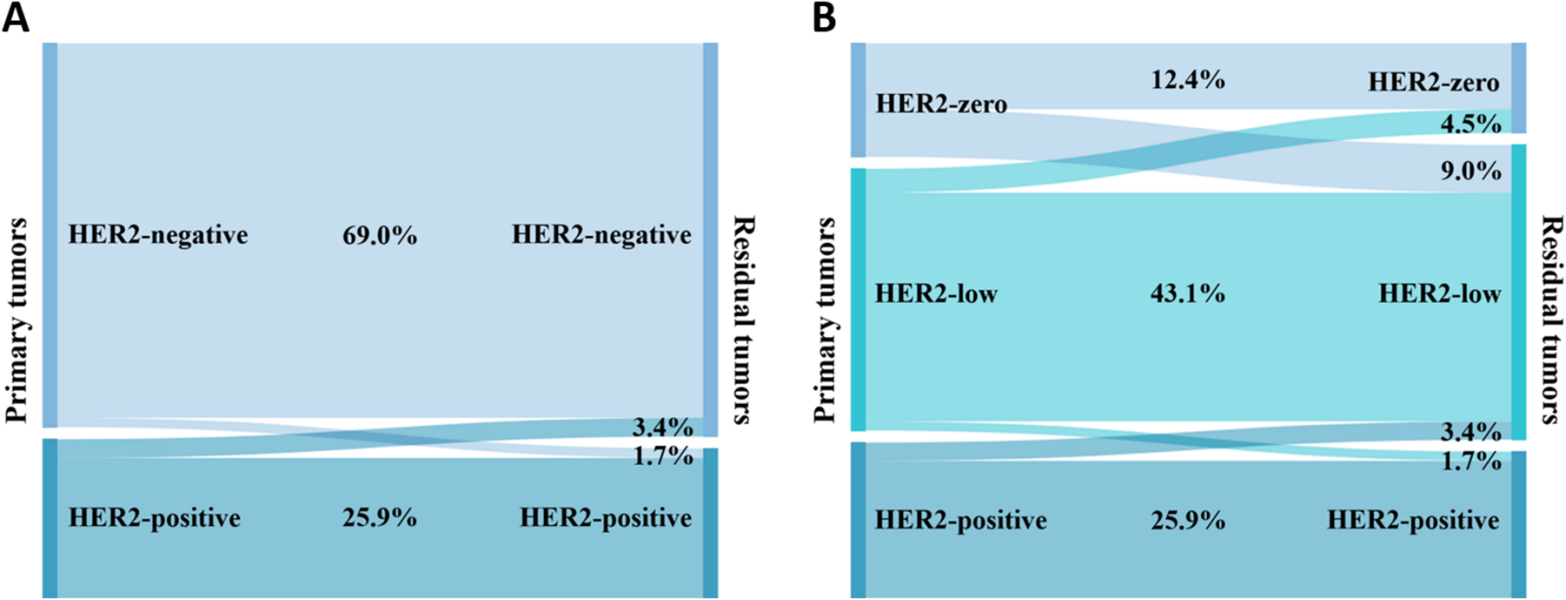
Conversion of HER2 expression between primary and residual tumors: **(A)** HER2 is categorized as negative or positive; **(B)** HER2 is categorized as zero, low, or positive.

**Figure 4A** shows the conversion of HER2 status in cases categorized as HER2-positive or HER2-negative. Of the primary breast cancers to residual lesions, HER2-negative converted to HER2-positive in 5 (1.7%) cases, and HER2-positive converted to HER2-negative in 10 (3.4%) cases. The rate of HER2 conversion was 5.1% (n = 15), and Cohen’s kappa coefficient was 0.873, indicating that the HER2 status was highly consistent.

**Figure 4B** shows the conversion of HER2 status in cases categorized as HER2-zero, HER2-low, or HER2-positive. After NAT, HER2-zero converted to HER2-low in 26 (9.0%) cases; HER2-low converted to HER2-zero in 13 (4.5%) cases, and HER2-low converted to HER2-positive in 5 (1.7%) cases; HER2-positive was converted to HER2-low in 10 (3.4%) cases; and no conversion was seen between HER2-zero and HER2-positive. The rate of HER2 conversion was 18.6% (n = 54), and Cohen’s kappa coefficient was 0.694, indicating that the consistency of HER2 status was relatively high.

**Figure 5:**
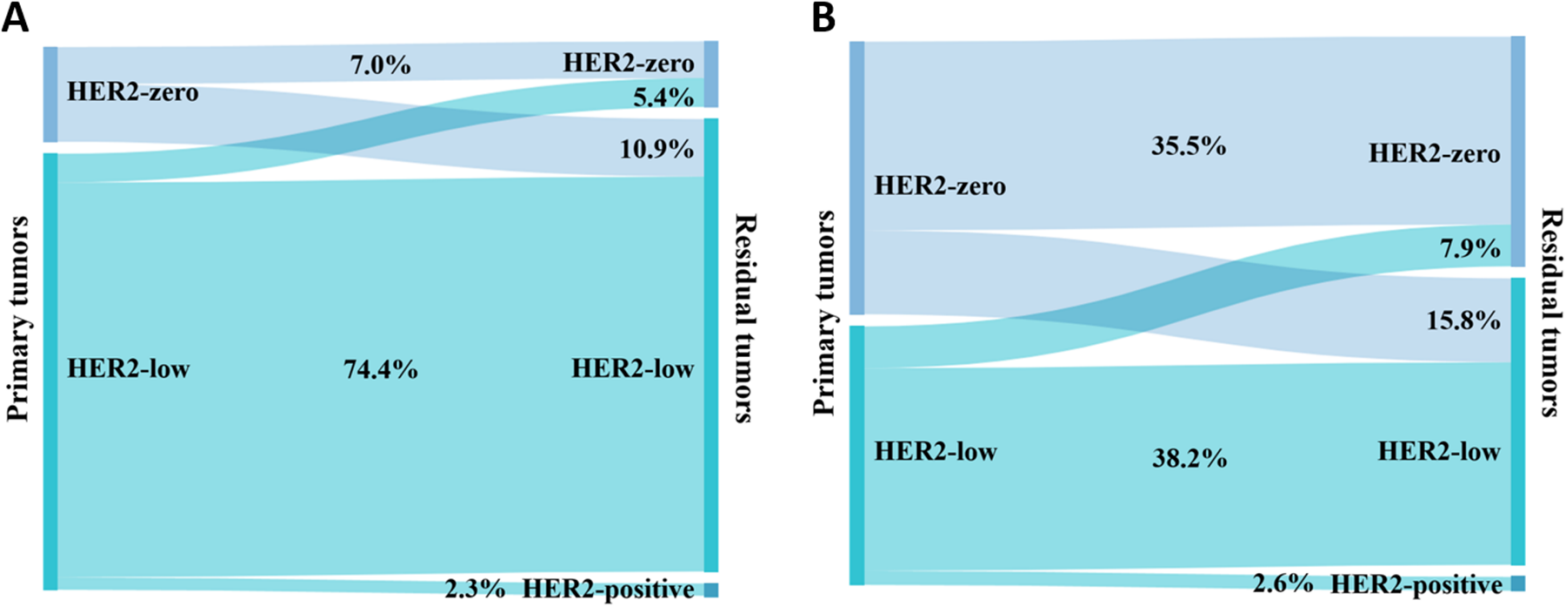
Conversion of expression in HER2-negative patients between primary and residual tumors: **(A)** Luminal and **(B)** TNBC.

There were 205 HER2-negative patients before NAT, comprising 129 (62.9%) with Luminal cancer and 76 (37.1%) with TNBC. **Figure 5** depicts the conversion of HER2 expression in the HER2-negative group based on the breast cancer phenotype. 24 (18.6%) patients with primary breast cancer of the Luminal type had a conversion of the HER2 status of the residual disease (**Figure 5A**). Of these, 14 (10.9%) converted from HER2-zero to HER2-low, 7 (5.4%) converted from HER2-low to HER2-zero, and 3 (2.3%) converted from HER2-low to HER2-positive. As shown in **Figure 5B**, 20 (26.3%) patients whose primary breast cancer was TNBC showed HER2 status conversion in residual tumors. 12 (15.8%) HER2-zero patients converted to HER2-low, 6 (7.9%) HER2-low patients converted to HER2-zero, and 2 (2.6%) HER2-low patients converted to HER2-positive. Patients with TNBC had a higher rate of HER2 conversion than those with the Luminal type; however, the difference was not statistically significant (*χ^2^* = 1.687, *P* = 0.194).

### 3.5 Clinicopathological factors associated with ER, PR, and HER2 conversion

**Table 4** shows clinicopathologic factors associated with ER, PR, and HER2 conversions. ER conversion correlated with pre-NAT HER2 status (*χ^2^* = 11.527, *P* = 0.003), with HER2-positive individuals having a higher likelihood of undergoing ER conversion. HER2 conversion correlated with pre-NAT HER2 status (*χ^2^* = 28.312, *P* < 0.001), with HER2-zero patients showing a higher likelihood of HER2 conversion. We found no clinicopathologic features associated with PR conversion.

**Table 4:** Clinicopathological factors associated with ER, PR, and HER2 conversion.

Factors	ER conversion				PR conversion				HER2 conversion			
	Yes	No	*χ²*/*F*	*P*	Yes	No	*χ²*/*F*	*P*	Yes	No	*χ²*/*F*	*P*
Age (years)			0.851	0.356			0.353	0.552			0.064	0.800
<50	10	146			23	133			23	105		
≥50	16	159			30	145			31	131		
Menopausal status			1.170	0.279			0.285	0.594			0.384	0.535
Pre-menopausal	10	151			24	137			27	107		
Post-menopausal	16	154			29	141			27	129		
Pretreatment tumor size(cm)			0.802	0.666			5.916	0.052			1.378	0.502
≤2	1	28			7	22			4	22		
2~5	19	220			31	208			37	173		
≥5	6	57			15	48			13	41		
Lymph node metastasis			0.208	0.648			0.864	0.353			0.013	0.908
Yes	20	222			36	206			40	173		
No	6	83			17	72			14	63		
Pretreatment ER status			1.563	0.211			0.042	0.838			1.126	0.289
Negative	7	120			21	106			26	95		
Positive	19	185			32	172			28	141		
Pretreatment PR status			0.077	0.781			0.412	0.521			0.171	0.680
Negative	10	109			17	102			22	89		
Positive	16	196			36	176			32	147		
Pretreatment HER2 status			11.527	0.003			1.324	0.516			28.312	<0.001
Zero	2	63			8	57			26	36		
Low	8	152			25	135			18	125		
Positive	16	90			20	86			10	75		
Pretreatment Ki-67			1.095	0.491			0.325	0.569			0.012	0.913
<15%	1	31			4	28			5	23		
≥15%	25	274			49	250			49	213		
Anti-HER2 targeted therapy			2.069	0.150			0.002	0.961			3.744	0.053
Yes	11	88			16	83			9	70		
No	15	217			37	195			45	166		

## 4 Discussion

In our cohort of 589 breast cancer patients receiving NAT, comprehensive analysis revealed significant correlations between baseline HR and HER2 expression levels with pathological treatment response. Notably, receptor status conversion was observed in a subset of patients following NAT completion. pCR is an alternative endpoint to disease-free survival (DFS) and OS in clinical studies of NAT in breast cancer and is critical to NAT efficacy (16). In our study, the pCR rates of patients with HER2-zero, -low, and -positive were 33.7%, 15.4%, and 57.0%, respectively, which are similar to the results of Zhang et al. (17). We observed a relatively low pCR rate in patients with TNBC, which may be associated with our treatment strategy (18). The NAT regimen in our study primarily consisted of anthracycline plus taxane-based therapy with or without cyclophosphamide, while published evidence demonstrates that the addition of platinum-based agents or immunotherapy can significantly improve pCR rates (19). In clinical practice, HER2-negative tumors receive only neoadjuvant endocrine therapy or chemotherapy, not anti-HER2 targeted therapy, and thus, HER2-negative patients exhibit the lowest pCR rate. HER2-low breast cancers are predominantly HR-positive, and hence, the distribution of molecular subtypes results in different pCR rates between HER2-zero and HER2-low (16). Several retrospective studies have shown that the percentage of ER- and PR-negative breast cancer patients receiving NAT who achieve pCR after chemotherapy is significantly higher than that of ER- and PR-positive breast cancer patients (20, 21). Our study showed similar results. This phenomenon may be explained by the fact that ER- and PR-negative breast tumors are poorly differentiated, have high dividing and proliferative activity, and are consequently more sensitive to chemotherapeutic agents (16). Molecular typing of breast cancers based on receptor status showed a pCR rate of 57.0% for HER2-positive breast cancers, which is similar to the results of recent studies (22, 23) but slightly higher than the result of a previous study (11). The higher pCR rate may be attributed to the ability of neoadjuvant targeted therapy containing trastuzumab to increase the pCR rate of HER2-positive breast carcinoma compared with conventional extra NAT (24). Moreover, we observed a lower pCR rate in the HER2+/HR+ cohort compared to the HER2+/HR− group. From a biological perspective, this may be attributed to bidirectional crosstalk between the HR and HER2 signaling pathways, which can contribute to resistance against both HER2-targeted therapies and endocrine treatments (25).

Our results revealed that the ER and PR conversion rates in residual lesions following NAT were 7.8% and 16.0%, respectively, which are comparable with the results of He et al. (26). The proportion of ER and PR undergoing conversion varies considerably across studies. A review of 32 publications showed that the HR status conversion rate between pre-NAT CNB specimens and post-NAT surgical specimens ranged from approximately 8% to 33%, the ER status conversion rate from approximately 2.5% to 17.0%, and the PR status conversion rate from approximately 5.9% to 51.7% (27). Several studies have shown that PR status is more likely to undergo conversion after NAT compared with ER (8, 10, 11, 26). This phenomenon is explained by the fact that PR expression often depends on intact signaling pathways, and therefore, PR exhibits a more heterogeneous spread within tumor cells (28).

There are no uniform conclusions about the factors influencing the conversion of ER and PR statuses. In a multifactorial logistic regression analysis by Yilmaz et al. (29), lower ER expression and smaller tumor size were found to be independent influences on ER and PR conversions, respectively. We found that pre-NAT HER2 status was an influential factor in ER conversion and did not find a correlation between clinicopathologic features and PR conversion. A study by Colleoni et al. (17) concluded that there was no significant association between conversions in HR status and clinicopathological characteristics of patients. The mechanisms by which conversions in HR status occur after NAT are complex. CNB and surgical resection biopsies are commonly considered to be highly concordant in detecting ER and PR expression (30). Possible reasons for changes in HR expression include fewer tumor cells in the CNB sample, which may not fully reflect the microenvironment inside the tumor, and technical problems in the assay. ASCO/CAP analyzed factors affecting receptor conversion, such as specimen handling, tissue fixation, and analytical assay methods; used 1% as the optimal cutoff for ER/PR positivity; and recommended that endocrine therapy for this subset of breast cancer patients could help mitigate biomarker changes and their possible adverse effects (31–33). In addition to this, Zhang et al. (34) showed that cases receiving NAT had a significantly higher incidence of discordant pre- and postoperative ER and PR statuses than cases not receiving NAT, implying that NAT drug administration may result in receptor status conversion. Sensitivity of tumor cells to chemotherapy correlates with HR status. HR-negative tumor cells are more sensitive to chemotherapy than HR-positive tumor cells, and therefore, tumor cells in residual lesions predominantly show HR positivity (35). Another explanation for the change from negative to positive HR could be due to the cells initially originating from HR-positive breast carcinoma cells and returning to their previous state under the influence of chemotherapy. In contrast, the conversion from positive to negative HR may be because the chemotherapy suppresses ovarian function, decreases circulating hormone levels, and downregulates ER and PR expression levels in premenopausal women (27). The different directions of HR conversion lead to prognostic differences in patients. Patients who converted to positive HR and received adjuvant endocrine therapy achieved better DFS and OS than HR-negative patients (36). Tacca et al. (35) analyzed the variations in ER and PR expression and their influence on survival and showed that the receptor status of the residual lesion influences the patient’s prognosis, not the subtype assessed at the time of the first biopsy. Therefore, it is clinically important to reassess the ER and PR statuses of the specimen after NAT. In particular, endocrine therapy is essential for HR-negative patients converted to HR-positive.

When we categorized HER2 as negative versus positive, the HER2 conversion rate was 5.1%, and decreased HER2 expression was more common than increased HER2 expression. It has been proposed that anti-HER2 targeted medications may be the cause of the lack of HER2 expression following NAT (11). Ignatov et al. (37) reported that 47.3% of HER2 conversion was from positive to negative with trastuzumab in NAT, and when the trastuzumab combined with the pertuzumab regimen was used, the rate of HER2 conversion from positive to negative increased to 63.2%. Here, we show that HER2 conversion was only connected with HER2 status in pre-NAT lesions and that individuals who had HER2-zero expression were most likely to undergo HER2 conversion; a similar conclusion was reported by Bo et al. (8). In addition, the HER2 conversion may be associated with tumor heterogeneity, inter- and intra-observer variability, variability in tissue handling and fixation, and sampling error (38, 39). The prognostic significance of HER2 expression variations is uncertain. A study reported that patients with absent HER2 status had worse DFS than those for whom HER2 status remained positive after NAT (40). In contrast, Yoshida et al. (41) performed a retrospective analysis and showed that variations in HER2 expression were not associated with patient prognosis.

The development of antibody-drug conjugates has brought HER2-low breast cancers to the forefront of research (42). The HER2 conversion rate before and after NAT increased to 18.6% when we included HER2-low in the subgroup. Shang et al. (43) demonstrated that the HER2 conversion rate was 21.42%, and this phenomenon was mainly caused by the conversion between HER2-zero and HER2-low. The results of Miglietta et al. (44) confirmed the instability of HER2-low in various environments. In the HER2-negative cohort, the rate of HER2 conversion was greater in TNBC than in Luminal breast cancer, but this difference was not statistically significant, which is comparable to the findings of Shang et al. (43).

As the conversion from HER2-zero to HER2-positive between pre-NAT primary breast cancers and post-NAT residual lesions is uncommon, and little has been learned about the clinical implications of the change in HER2 status after NAT, the present standard of treatment is determined by the receptor expression of the patient’s primary breast cancer (45). A study of the relationship between repeat biopsies and HER2-low presented at ASCO 2023 found that the percentage of cases with HER2-low expression increased alongside the number of repeat biopsies, with 59%, 73%, 83%, 83%, and 100% of cases with HER2-low expression at 1, 2, 3, 4, and ≥5 biopsies, respectively (46). In conjunction with the existing literature and the results of this study, it is significant to re-examine the HER2 status of post-NAT samples to better assess which patients may experience a conversion from HER2-zero to HER2-low, as well as informing clinical trials for patients with low HER2 so that these patients can be treated with novel drugs that may be effective.

This research has several noteworthy limitations. First, the single-center retrospective design, coupled with a relatively small sample size and incomplete follow-up data, precluded robust survival analysis. Second, the study protocol did not systematically evaluate the correlation between neoadjuvant therapeutic regimens and receptor status conversion, which constrains the clinical applicability of our findings. Finally, selection bias was inevitable as our HER2 conversion analysis was restricted to patients with definitively documented HER2 status.

## 5 Conclusion

Some breast cancer patients may have conversions of ER, PR, and HER2 status after NAT. Residual lesions must be immunohistochemically re-evaluated to determine the patient’s receptor expression status and adjust the future therapy plan.

## Data Availability

The original contributions presented in the study are included in the article/supplementary material. Further inquiries can be directed to the corresponding authors.
